# Supplementary material for: Temperate Zone Plant Natural Products—A Novel Resource for Activity against Tropical Parasitic Diseases
Source: Pharmaceuticals (Basel). 2021 Mar 7;14(3):227. doi: 10.3390/ph14030227 (PMC7998250; doi:10.3390/ph14030227)
Supplement: Supplementary file 1 [file pharmaceuticals-14-00227-s001.zip › Supplementary Files/Figures S1-4.docx]

Article

Supplementary Figures

Temperate zone plant natural products – a novel resource for activity against tropical parasitic diseases

Hamza Hameed ^1,2,#^, Elizabeth FB King ^1,#^, Katerina Doleckova ^1,3^, Barbara Bartholomew ^4^, Jackie Hollinshead ^4^, Haddijatou Mbye ^1,5^, Imran Ullah ^1,6^, Karen Walker ^7^, Maria Van Veelen ^1^, Somaia Saif Abou-Akkada ^8^, Robert J Nash ^4^, Paul D Horrocks ^1*^ and Helen P Price ^1,*^

^#^ These authors contributed equally to this publication

^1^ Centre for Applied Entomology and Parasitology, Keele University, Staffordshire, UK; [e.f.b.king@keele.ac.uk](mailto:e.f.b.king@keele.ac.uk), [h.price@keele.ac.uk](mailto:h.price@keele.ac.uk), [p.d.horrocks@keele.ac.uk](mailto:p.d.horrocks@keele.ac.uk), [w4r96@students.keele.ac.uk](mailto:w4r96@students.keele.ac.uk)

^2^ Department of Chemistry , College of Education For Pure Science , University of Mosul, Mosul, Iraq; hamza83n@uomosul.edu.iq

^3^ Department of Biology, Faculty of Life Sciences, University of Hradec Králové, Czech Republic; [k.doleckova@centrum.cz](mailto:k.doleckova@centrum.cz)

^4^ PhytoQuest Limited, Aberystwyth, UK; [robert.nash@phytoquest.co.uk](mailto:robert.nash@phytoquest.co.uk), [barbara.bartholomew@phytoquest.co.uk](mailto:barbara.bartholomew@phytoquest.co.uk), [jackie.hollinshead@yahoo.com](mailto:jackie.hollinshead@yahoo.com)

^5^ MRC Unit The Gambia at LSHTM, Atlantic Boulevard, Fajara, Banjul, The Gambia; [haddijmbye@gmail.com](mailto:haddijmbye@gmail.com)

^6^ Harvard T.H. Chan School of Public Health, Harvard University, Boston, MA, USA; [iullah@hsph.harvard.edu](mailto:iullah@hsph.harvard.edu)

^7^  School of Life Sciences, Keele University, Staffordshire, UK; [kwalker56789@gmail.com](mailto:kwalker56789@gmail.com)

^8^ Faculty of Veterinary Medicine, Alexandria University, Egypt; [somaia_abuakkada@yahoo.com](mailto:somaia_abuakkada@yahoo.com)

***** Correspondence: [h.price@keele.ac.uk](mailto:h.price@keele.ac.uk); Tel.: +44-1782-734219 (H.P.P.), [p.d.horrocks@keele.ac.uk](mailto:p.d.horrocks@keele.ac.uk); Tel.: +44-1782-734732 (P.D.H.).

Received: date; Accepted: date; Published: date

**Figure S1**: Structures of compounds of all initial hits identified in Figure 1 and reported in the text of this study. SMILES codes are available for the initial hits in Table S2.


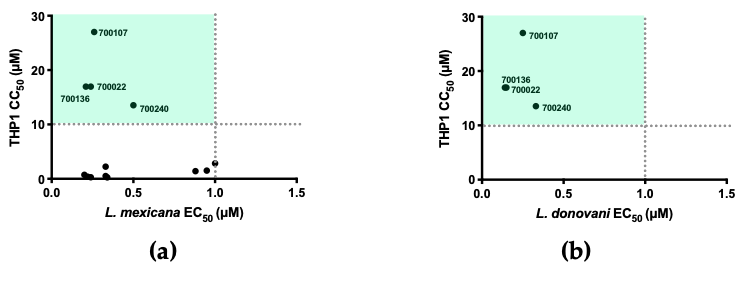


**Figure S2**: Comparison of *Leishmania spp*. potency and cytotoxicity against THP1 of the initial hit compounds. Comparison of the EC_50_ of hit compounds in **A** *L. mexicana*, **B** *L. donovani* to the CC_50_ of the same compounds in the THP1 cell line*.* The grey dotted lines indicate the preferred potency (EC_50_<1μM) and THP1 CC_50_ (>10μM to provide a minimum SI≥10), with hits taken forward from the top left quadrant only.


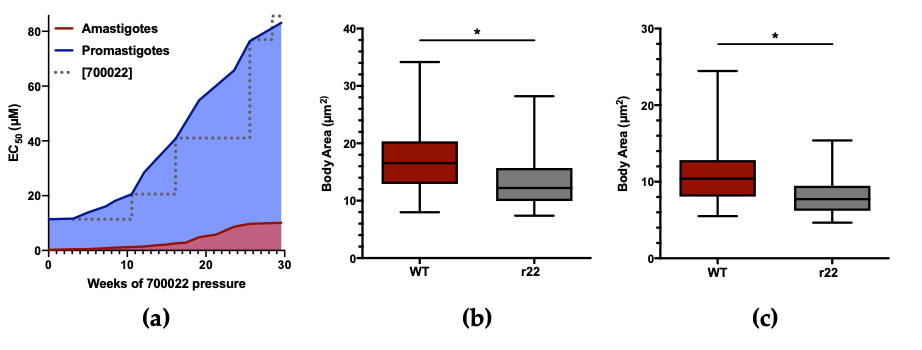


**Figure S3**: *L. mexicana* 700022 resistant line**.** **A** The increase in *L. mexicana* resistance of 700022 seen over the 28 weeks of drug pressure. Blue shows promastigote EC_50_ and red shows amastigote EC_50_. The grey line show the concentration of 700022 being applied. **B** Body size comparisons between WT and r22 promastigotes (285 parasites of each) were made by analysing fluorescence microscopy images using ImageJ. Statistical significance was assessed using the Mann-Whitney U test was performed; the p-value<2.2×10^-16^ confirmed statistical difference. **C** Cell body area comparisons between WT and r22 axenic amastigotes (148 and 136 parasites, respectively) were made by analysing fluorescence microscopy images using ImageJ. Statistical significance was assessed using the Mann-Whitney U test was performed; the p-value=6.379×10^-16^ confirmed statistical difference.

**Figure S4**: *Leishmania* intramacrophage assays. **A** *L. mexicana* infected macrophages following exposure to 1×, 3× and 9× EC_50_ of each of the anti-leishmanial sterols or amphotericin B. The data shows a concentration dependent effect across the sterols, although this activity is less than that of amphotericin B The white box reports data where no compound or drug is applied. Data represents the mean and standard deviation (n=4). **B** Average number of parasites per macrophage in the same experiment as **A**. A Kruskal-Wallis test followed by Dunn’s multiple comparison test was used to determine a significant difference (p<0.05) of each treatment to the untreated control and have been indicated with those being significant marked with an asterix. **C** Comparing 700022 activity between axenic amastigotes (red) and intramacrophage amastigotes (blue). Concentration–response curves for compound 700022 against: intracellular *L. mexicana* NanoLuc-PEST-transgenic line (blue), axenic amastigotes of NanoLuc-PEST-transgenic line (red) as well as the human cell lines HepG2 (green) and THP-1 (black). The data for the intracellular *L. mexicana* NanoLuc-PEST-transgenic line represent one biological repeat of three technical repeats.

| 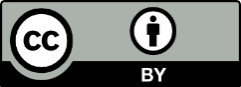 | © 2020 by the authors. Submitted for possible open access publication under the terms and conditions of the Creative Commons Attribution (CC BY) license (http://creativecommons.org/licenses/by/4.0/). |
| --- | --- |
