# Supplementary figures and images for: Temperate Zone Plant Natural Products—A Novel Resource for Activity against Tropical Parasitic Diseases
Source: Pharmaceuticals (Basel). 2021 Mar 7;14(3):227. doi: 10.3390/ph14030227 (PMC7998250; doi:10.3390/ph14030227)

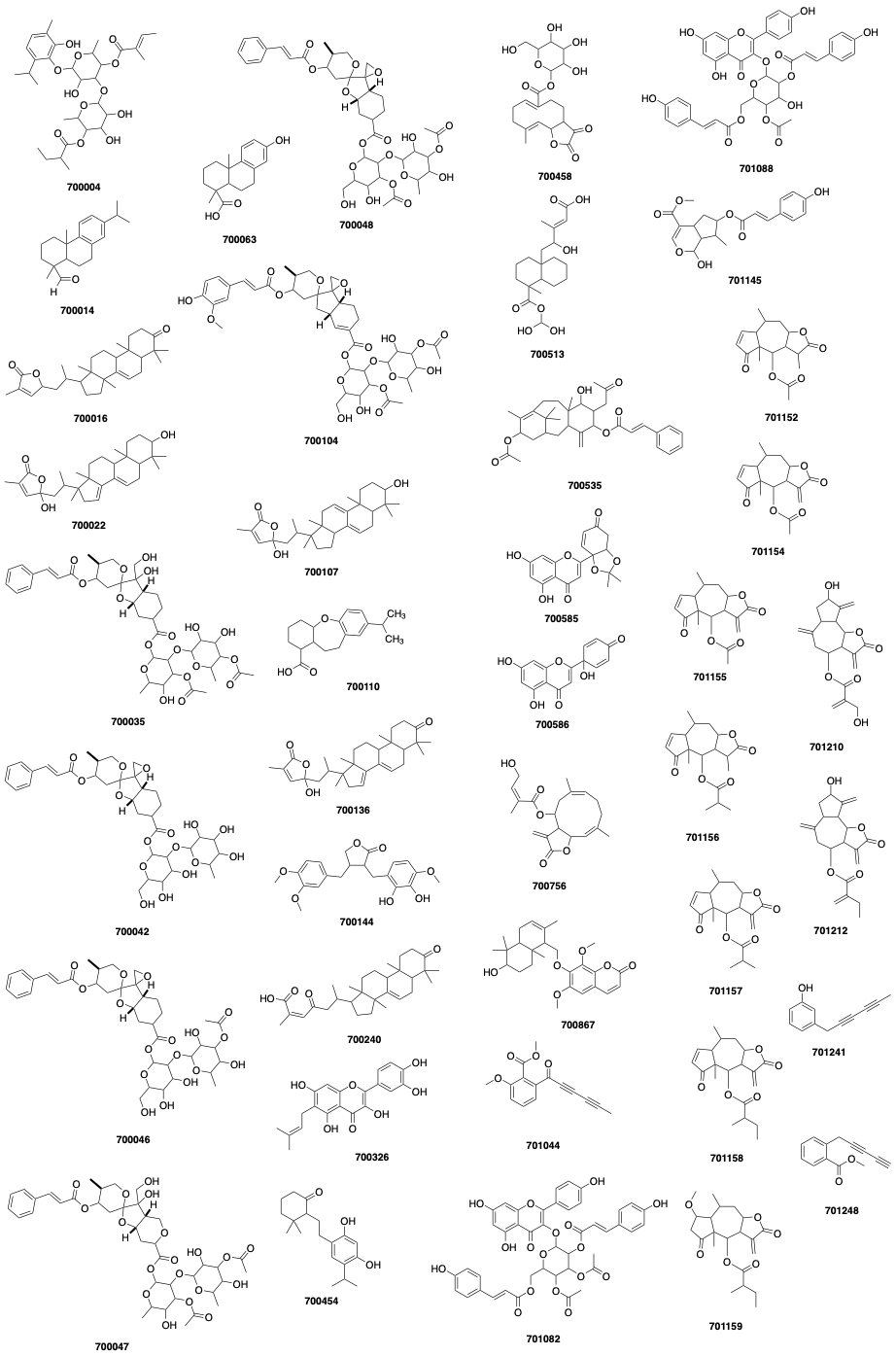

Supplement: Supplementary file 1 [file pharmaceuticals-14-00227-s001.zip › Supplementary Files/Figure S1.png]

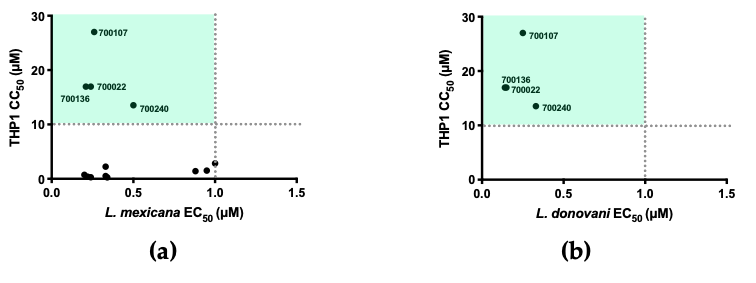

Supplement: Supplementary file 1 [file pharmaceuticals-14-00227-s001.zip › Supplementary Files/FIgure S2.png]

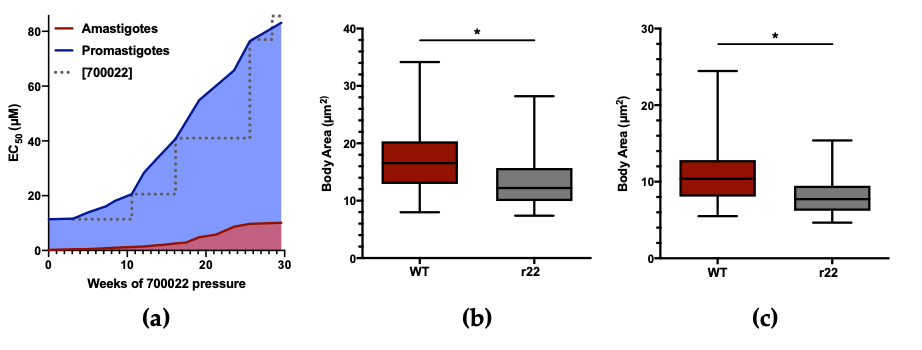

Supplement: Supplementary file 1 [file pharmaceuticals-14-00227-s001.zip › Supplementary Files/Figure S3.png]

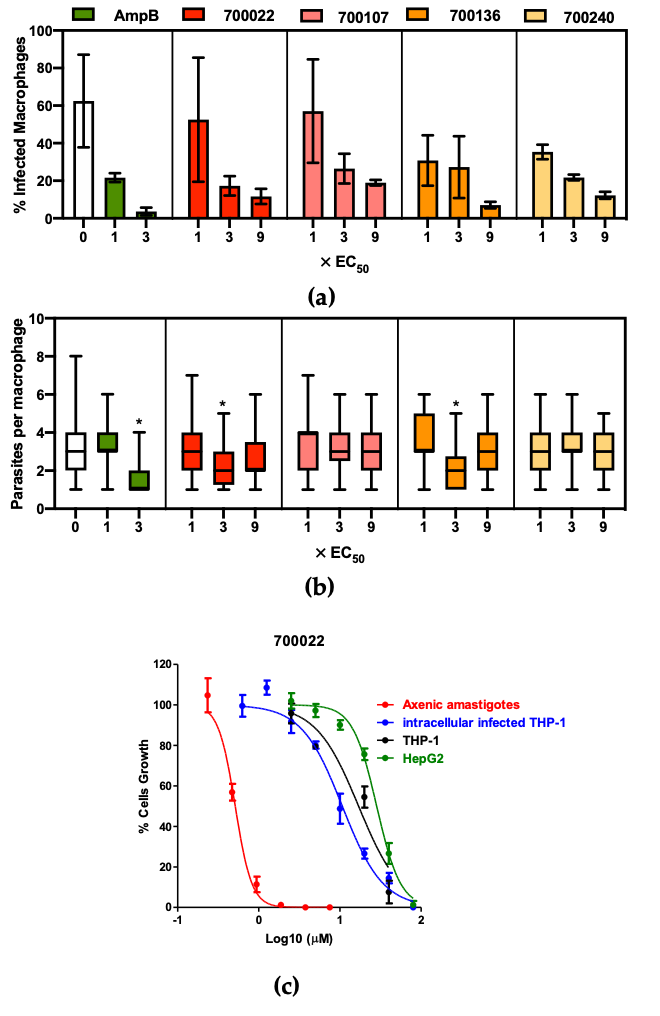

Supplement: Supplementary file 1 [file pharmaceuticals-14-00227-s001.zip › Supplementary Files/Figure S4.png]
